# A digital twin and deep-learning ensemble for cyber attack detection in industrial control systems at the IoT edge

**DOI:** 10.1038/s41598-026-53863-z

**Published:** 2026-05-22

**Authors:** Ali Sayghe, Mohammad D. Alahmadi, Abdulrahman A. Gharawi

**Affiliations:** 1 Department of Electrical Engineering, Yanbu Industrial College, Yanbu, Saudi Arabia; 2 Department of Software Engineering, College of Computer Science and Engineering, University of Jeddah, Jeddah, Saudi Arabia; 3 Department of Computer Science, University College of Al Jamoum, Umm Al-Qura University, Makkah, Saudi Arabia

**Keywords:** Digital twin, Industrial control systems, Cyber-physical security, Anomaly detection, Edge computing, AI-driven cyber threats, Deep learning, Industrial IoT, Engineering, Mathematics and computing

## Abstract

Industrial Control Systems (ICS) face escalating cyber threats as adversaries increasingly exploit artificial intelligence (AI) to evade conventional defenses. This paper introduces a Digital Twin-enhanced security framework in which a real-time, physics-consistent virtual replica of the controlled industrial process is synchronized with sensor and actuator telemetry from the physical plant and used to validate, suppress, or confirm anomaly scores produced by a deep-learning ensemble. The physical twin is the closed-loop ICS plant (water treatment, water distribution, or chemical process); the Digital Twin is a state-space process model coupled to an Extended Kalman Filter that predicts the next sensor measurement and emits a residual whenever the observation deviates from the physics-consistent prediction. The detection layer combines this Digital-Twin residual signal with a Long Short-Term Memory (LSTM) autoencoder, an attention-based transformer, and an Isolation Forest, fused through a calibrated weighted score that is gated by the residual, so that purely data-driven anomalies that do not violate physics are downweighted and stealthy attacks that violate physics are escalated. Evaluated on three benchmark datasets (Secure Water Treatment testbed [SWaT], Water Distribution [WADI], and Tennessee Eastman) comprising 56 attack scenarios, the framework achieves 97.6% precision, 96.2% recall, an F1-score of 96.9%, and sub-50 ms inference latency. This corresponds to a 3.2 percentage-point F1-score improvement over the strongest baseline (transformer at 93.7%) and a roughly 50% reduction in residual error. Interpretability is supported through attention visualization and Digital-Twin residual analysis, enabling operators to validate detection outcomes. With native Message Queuing Telemetry Transport (MQTT) and Open Platform Communications Unified Architecture (OPC UA) integration, Byzantine fault-tolerant consensus for distributed deployments, and formal verification of safety properties, the framework supports deployment-oriented protection for critical infrastructure aligned with International Electrotechnical Commission (IEC) 62443-4-2 requirements.

## Introduction

The convergence of Operational Technology (OT) and Information Technology in modern ICS has created unprecedented cybersecurity vulnerabilities^1,2^. With Industrial IoT deployments projected to reach 75 billion devices by 2025^3^, distributed architectures exponentially increase attack surfaces while legacy protocols lack modern security features. Traditional IT security tools fail in OT environments due to differing operational requirements^4^, while purely physics-based approaches achieve only about 88.6% accuracy compared with around 96% for hybrid methods^5,6^. Machine-learning approaches reach higher accuracy but suffer from high false-positive rates and a lack of interpretability^7,8^. Current Digital Twins focus on operations and maintenance rather than security, with only 23% incorporating security from inception^9^. Edge-computing constraints further complicate deployment, with lightweight convolutional neural networks (CNNs) sacrificing accuracy for efficiency^10^.

This paper presents a Digital Twin-enhanced security framework that integrates physics-based modeling with deep learning for distributed industrial environments. We use the term *Digital Twin* in a strict, technical sense: a continuously updated virtual model of a specific physical ICS process whose internal state is synchronized in real time with sensor and actuator telemetry from the plant, and whose physics-consistent state predictions are used to generate residuals that validate, suppress, or escalate anomaly scores produced by a separate machine-learning ensemble. The *physical twin* is the operating ICS plant itself—the chain of tanks, valves, pumps, and reactors instrumented in SWaT, WADI, and the Tennessee Eastman challenge. The Digital Twin is therefore not a metaphor for “the security system” nor a label attached to a generic simulation model; it is the synchronized state estimator and process model that produces measurable, falsifiable predictions of the plant’s behavior at every sampling step.

The framework introduces a hybrid architecture that integrates MATLAB/Simulink physics models with Python-based deep learning, achieving 25.4 ms end-to-end latency while combining interpretability with adaptability. The ensemble detector combines LSTM autoencoders, attention-based anomaly transformers, and Isolation Forests, achieving 97.6% precision with interpretable attention visualization. The framework provides broad attack coverage, detecting FDI (97.2% accuracy), replay attacks (94.8%), and stealthy attacks designed to evade traditional intrusion detection systems. Deployment-oriented implementation includes MQTT/OPC UA integration, Byzantine fault tolerance, and formal verification guidelines aligned with IEC 62443-4-2 requirements^11^. The framework achieves 68.3% detection against adaptive attacks, compared with 28.4% for baselines, contributing to progress in defender-attacker dynamics.

Digital Twins have evolved significantly from operational tools to security instruments for cyber-physical systems^12^. The concept of Security-Enhancing Digital Twins (SEDTs) demonstrated that Digital Twins could provide safe environments for vulnerability assessment without operational disruption^13^. Recent implementations achieve high synchronization rates, with frameworks maintaining 100 Hz synchronization with SCADA systems while performing continuous anomaly detection^14^. The integration of blockchain technology has addressed trust and integrity challenges^15^. The proliferation of Industrial IoT devices has fundamentally changed the security landscape, with edge computing offering local processing capabilities^10,16^. Deep-learning approaches have shown promise for ICS anomaly detection, with RNNs achieving 93% accuracy^17^, LSTM networks reaching 95%^18^, and transformer-based methods achieving 96% accuracy^19^. Multi-Scale Dynamic Graph Neural Network architectures achieve F1-scores of 0.8886^20^, while Dual Attention Networks show 49.4% improvement through attention mechanisms^21^. The sophistication of attacks targeting ICS has increased dramatically: FDI attacks exploit temporal correlations^22,23^; replay attacks blind security systems^5,24^; and stealthy attacks exploit system nullspaces^25,26^. AI-driven attack generation with large language models can generate exploit code with a 67% success rate^27^. Our framework addresses these gaps by providing a Digital Twin-enhanced security framework specifically designed for distributed industrial IoT and edge computing environments, in which the Digital Twin contributes a verifiable physics-consistency check that purely data-driven detectors cannot supply.

The rest of this paper is organized as follows. We first review related work on Digital Twin security, ICS anomaly detection, and adversarial robustness, and present our contributions. We then describe the framework architecture, detection algorithms, and implementation, followed by experimental results across three benchmark datasets, a discussion of implications, deployment considerations, and limitations, and a conclusion summarizing contributions and future directions.

## Related work

Digital Twins have evolved from operational optimization tools to security instruments for cyber-physical systems. Eckhart et al^13^. introduced SEDTs providing safe vulnerability assessment environments without operational disruption. Sayghe^14^ achieved 100 Hz SCADA synchronization for continuous anomaly detection with 96% accuracy. Blockchain integration has addressed trust challenges in distributed Digital Twins^15^. However, these approaches focus on isolated components rather than integrated security frameworks with formal verification guarantees, real-time edge deployment constraints, and an explicit residual-based mechanism by which the Digital Twin influences the detection decision.

ICS anomaly detection methods span three paradigms. Physics-based approaches using state estimation and constraint checking achieve 88–92% accuracy^5^ but struggle with complex non-linear dynamics and produce high false-positive rates in noisy industrial environments. Machine-learning methods reach higher accuracy: RNNs 93%^17^, LSTMs 95%^18^, and transformers 96%^19^, but lack interpretability—critical for safety-critical systems—and are vulnerable to adversarial perturbations. Recent hybrid approaches combining graph neural networks with physics models achieve 95.8% accuracy^28^, yet do not address real-time edge deployment (45 ms latency) or provide formal verification guarantees essential for regulatory approval.

Adversarial robustness in ICS remains challenging. Jedrzejewski et al^23^. surveyed adversarial machine learning in industry, finding 67% of defense mechanisms fail against adaptive attacks specifically designed to evade detection. Cohen et al^24^. introduced certified robustness via randomized smoothing, providing provable guarantees, but their work focused on computer vision rather than ICS time-series data. No prior work adapts certified robustness to the multivariate temporal dependencies and physical constraints inherent in industrial control systems.

Byzantine fault tolerance has been extensively studied for distributed systems^29^, providing resilience against up to $f = \lfloor (n-1)/3\rfloor$ malicious nodes. However, prior work does not address real-time ICS constraints where control loops operate at 100–1000 ms intervals, requiring consensus latency below 50 ms. Edge computing for ICS security^16^ achieves 45 ms response time but lacks Byzantine fault tolerance, while Byzantine consensus implementations^29^ do not provide formal latency bounds suitable for safety-critical applications.

Standards compliance for ICS security, particularly IEC 62443^11^, NIST SP 800-82, and ISO 27001, is essential for regulatory approval and deployment in critical infrastructure. However, most existing work claims compliance without rigorous verification. No prior work provides systematic item-level compliance mapping with assessment criteria and test procedures necessary for third-party certification.

Our framework advances beyond this state-of-the-art by: (1) integrating Digital Twins with multi-modal ML ensembles through formal state synchronization and an explicit residual-based fusion gate, achieving 97.6% precision versus 95.8% in the best prior work; (2) adapting certified robustness to ICS temporal data, with 78.6% versus 42.3% detection against FGSM attacks; (3) providing deployment-oriented Byzantine consensus with sub-50 ms latency and graceful degradation; and (4) systematic IEC 62443 compliance mapping with 90% Security Level 2 coverage versus unsubstantiated prior claims.

### Novel contributions

This work makes the following contributions advancing ICS security:

**1. Integrated Digital Twin-Enhanced Ensemble Architecture.** We present a security framework integrating a physics-based Digital Twin with a multi-modal ML ensemble (LSTM + Transformer + Isolation Forest) through formal state synchronization. Unlike prior work using physics^5^ and ML^19^ separately, or hybrid approaches^28^ lacking an explicit synchronization mechanism, our Digital Twin provides a common state representation that enables residual-based validation of learned detectors (Algorithm 1, Eqs. 1–11). This achieves 97.6% precision versus 95.8% in the best prior work, while maintaining interpretability through attention visualization (a 3.2 percentage-point F1-score improvement over the strongest baseline).

**2. Certified Adversarial Robustness for ICS Time-Series.** We adapt randomized smoothing^24^ from computer vision to ICS temporal data, providing certified robustness guarantees for industrial anomaly detection. Our temporal smoothing with autoregressive noise model (Eq. 17) achieves 78.6% versus 42.3% detection against FGSM attacks (a 36.3 percentage-point absolute improvement), with theoretical certification radius $\epsilon =0.05$ for the $L_\infty$ norm, and is validated by a 39.9 percentage-point improvement against adaptive attacks (Table 6).

**3. Deployment-Oriented Byzantine Fault Tolerance.** We provide a practical PBFT implementation for distributed ICS anomaly detection with formal latency bounds. Our time-bounded PBFT variant maintains 96.7% detection with 30% node failure and sub-50 ms consensus latency, in contrast to prior work^16^ achieving 45 ms without fault tolerance, enabling critical infrastructure deployment with the $n \ge 3f+1$ node requirement and provable safety properties.

**4. Systematic Standards Compliance Framework.** We provide a comprehensive IEC 62443-4-2 compliance mapping for ML-based ICS security with 90% Security Level 2 coverage (61 of 68 requirements fully met) including item-level assessment criteria and test procedures, in contrast to unsubstantiated prior compliance claims^28^. This enables regulatory approval and deployment in safety-critical applications.

## Methods

We organize the methodology so that each subsection feeds the next. We first define the physical twin (the controlled ICS plant) and its dynamics. We then formulate the Digital Twin and its synchronization with the physical plant, and describe the residual signal that emerges from this synchronization. The residual is then combined with three machine-learning detectors and fused through a calibrated, physics-consistency-gated voting rule. The downstream subsections cover attack classification and response, edge deployment with Byzantine consensus, and the experimental protocol used in the Results.

### System model and physical twin definition

We modeled an ICS as a multilayer cyber-physical system $\mathscr {S} = \langle \mathscr {P}, \mathscr {C}, \mathscr {N}, \mathscr {H}, \mathscr {I} \rangle$ where each layer represents distinct operational components with formal specifications and interdependencies. The *physical twin* is the physical process layer $\mathscr {P}$, which encompasses the actual industrial equipment under control: the multi-stage water-treatment train of SWaT, the water-distribution network of WADI, or the chemical reactor and separator network of the Tennessee Eastman process. Its hybrid dynamics combine continuous evolution with discrete state transitions and are written as

1 $$\begin{aligned} \begin{aligned} \dot{x}(t)&= f(x(t), u(t), w(t)), \quad x \in \mathscr {X} \subseteq \mathbb {R}^n, \\ y(t)&= g(x(t), v(t)) + \delta (t), \end{aligned} \end{aligned}$$

where *x*(*t*) is the system state, *u*(*t*) the control input, *w*(*t*) the process noise, and $\delta (t)$ encodes sensor anomalies including bias faults, drift, complete sensor failures, or adversarial injection. For discrete-time implementation with sampling period $T_s$, the system dynamics become

2 $$\begin{aligned} \begin{aligned} x_{k+1}&= A_d x_k + B_d u_k + G_d w_k, \\ y_k&= C_d x_k + D_d u_k + H_d v_k + \delta _k. \end{aligned} \end{aligned}$$

The control layer $\mathscr {C}$ implements feedback control algorithms with formal safety guarantees expressed in Linear Temporal Logic. The control law incorporates both reference tracking and state-estimation feedback with safety constraints, and is given by

3 $$\begin{aligned} u(t) = {\left\{ \begin{array}{ll} K(r(t) - y(t)) + F(\hat{x}(t)) & \text {if } \phi _{\text {safety}}, \\ u_{\text {safe}} & \text {otherwise}. \end{array}\right. } \end{aligned}$$

The network layer $\mathscr {N}$ incorporates Time-Sensitive Networking standards to provide deterministic communication guarantees essential for real-time control. The interdependency layer $\mathscr {I}$ models cascading failure propagation across system layers using multilayer network theory.

### Digital twin formulation and synchronization

What is being twinned. The Digital Twin is a synchronized virtual replica of the closed-loop ICS process described by Eqs. 1–2. For SWaT it twins the six-stage water-treatment train (raw water intake, chemical dosing, ultrafiltration, dechlorination, reverse osmosis, and product storage), with state $x_k \in \mathbb {R}^{51}$ corresponding to tank levels, flow rates, pressures, conductivities, pH, ORP, and valve/pump status. For WADI it twins the water-distribution network state $x_k \in \mathbb {R}^{123}$ over primary, elevated reservoir, and consumer-tank stages. For Tennessee Eastman it twins the reactor–separator–stripper loop with $x_k \in \mathbb {R}^{52}$ process variables. In every case, the Digital Twin is therefore tied to a specific, identifiable physical plant whose dynamics are governed by mass and energy balances, hydraulic relations, and discrete actuator logic; it is not a generic anomaly detector wrapped with the label “Digital Twin.”

Digital-Twin process model and predicted state. Let $\hat{x}_k$ denote the Digital-Twin internal state and $\hat{f}, \hat{g}$ the first-principles process model identified offline (MATLAB/Simulink for SWaT and WADI, the Downs–Vogel reactor model for Tennessee Eastman). The Digital Twin propagates its state as

4 $$\begin{aligned} \hat{x}_{k+1\mid k} = \hat{f}(\hat{x}_{k\mid k}, u_k), \qquad \hat{y}_{k+1\mid k} = \hat{g}(\hat{x}_{k+1\mid k}), \end{aligned}$$

where $\hat{y}_{k+1\mid k}$ is the physics-consistent prediction of the next sensor measurement.

Synchronization with the physical twin. At every sampling instant the Digital Twin is synchronized with the physical plant through a bounded Extended Kalman Filter (EKF) update that uses the actually observed measurement $y_k$ to correct $\hat{x}_k$:

5 $$\begin{aligned} \hat{x}_{k\mid k} = \hat{x}_{k\mid k-1} + K_k\,(y_k - \hat{y}_{k\mid k-1}), \quad K_k = P_{k\mid k-1} C_d^{\!\top }\!\left( C_d P_{k\mid k-1} C_d^{\!\top } + R\right) ^{-1}. \end{aligned}$$

This update runs at the SCADA telemetry rate (10 Hz on the evaluated datasets, with the protocol stack supporting up to 100 Hz on instrumented testbeds^14^). Synchronization is therefore not symbolic: the same measurements that enter the controller are simultaneously fed to the Digital Twin so that, under nominal operation, $\hat{x}_{k\mid k} \rightarrow x_k$ in mean square error within bounded covariance $P_{k\mid k}$.

Residual signal. The synchronized Digital Twin produces, at every step, an innovation residual

6 $$\begin{aligned} r_k = y_k - \hat{y}_{k\mid k-1} \end{aligned}$$

and a normalized Digital-Twin anomaly score based on its Mahalanobis norm under the residual covariance $S_k = C_d P_{k\mid k-1} C_d^{\!\top } + R$,

7 $$\begin{aligned} s_{\text {DT},k} = \tfrac{1}{m}\, r_k^{\!\top } S_k^{-1} r_k, \end{aligned}$$

where *m* is the number of monitored sensors. Under nominal conditions, $s_{\text {DT},k}$ is approximately $\chi ^2_m$-distributed; deviations therefore have a calibrated false-alarm rate that is independent of the learned ML components.

How the Digital Twin differs from a generic simulator. A generic simulator integrates Eq. 1 in open loop and never sees real measurements; it cannot detect attacks because there is no point of comparison. The Digital Twin defined by Eqs. 4–6 is closed by the synchronization step in Eq. 5, and it is precisely this closure that produces the residual $r_k$. Equally, a learned anomaly detector consumes $y_k$ but does not consume $u_k$ through a physical model, so it cannot tell whether an apparently anomalous reading is consistent with the actuators that were actually commanded. The Digital Twin couples the two streams.

Contribution to anomaly detection and what is lost without it. The Digital Twin contributes to detection in three concrete ways. First, $s_{\text {DT},k}$ is itself an anomaly statistic with calibrated false-alarm probability, used as one of the fused components (Eqs. 12–13). Second, $r_k$ is used as a physics-consistency *gate* on the ML score: when the ML ensemble flags a window but $r_k$ remains within its $\chi ^2$ control limits, the alarm is suppressed as a likely false positive (this mechanism removes a substantial fraction of the false alarms that would otherwise be raised by data-distribution shifts). Third, residuals expose stealthy null-space attacks^25^ that are designed to remain temporally normal: such attacks must drive $x_k$ off the manifold described by $\hat{f}$ and therefore cause $r_k$ to grow, even when no ML detector reacts. The ablation in Table 4 quantifies this contribution: removing the Digital-Twin/physics path drops the F1-score by 3.1 percentage points and disproportionately degrades stealthy- and replay-attack detection, because ML-only ensembles can no longer cross-check whether a learned “normal” pattern is also physically attainable.

### Physics-based residual generation

The residual signal of Eq. 6 is also augmented with Linear Temporal Logic (LTL) checks that encode plant safety properties: $\square (\lnot \,\text {unsafe\_state})$ guarantees the system never enters dangerous states (for example, tank overflow in SWaT or reactor-pressure violation in Tennessee Eastman), while $\square \diamond \,\text {response}$ ensures legitimate requests receive responses within bounded intervals. Whenever an LTL property is violated, the corresponding component of $r_k$ is flagged in $s_{\text {DT},k}$ and forwarded to the classification module.

### Machine-learning detection models

Three complementary algorithms provide diverse, data-driven anomaly detection in parallel with the Digital Twin. The LSTM autoencoder captures temporal dependencies through recurrent processing and learns to reconstruct normal operation sequences, flagging significant reconstruction errors as anomalies. Its hidden state and reconstruction follow

8 $$\begin{aligned} h_t = \textrm{LSTM}(x_t, h_{t-1}), \qquad \hat{x}_t = \textrm{Decoder}(h_t), \end{aligned}$$

where $h_t$ is the hidden state at time *t*, $x_t$ is the input sensor reading, and $\hat{x}_t$ is the reconstructed output. The anomaly score is computed as $\Vert x_t - \hat{x}_t\Vert _2$, exceeding a learned threshold whenever a temporal pattern deviates from training.

The attention transformer identifies spatial relationships between sensors using scaled dot-product attention, enabling detection of coordinated multi-sensor attacks through

9 $$\begin{aligned} \textrm{Attention}(Q,K,V) = \textrm{softmax}\!\left( \frac{QK^{\!\top }}{\sqrt{d_k}}\right) V, \end{aligned}$$

where *Q*, *K*, and *V* are query, key, and value projections of sensor readings. The attention weights reveal sensor importance for each prediction, providing interpretability through visualization of which sensors contribute to anomaly decisions.

The Isolation Forest rapidly detects outliers through path-length analysis in binary trees, isolating anomalies in fewer splits than normal points; its score is given by

10 $$\begin{aligned} s(x,n) = 2^{-\frac{E(h(x))}{c(n)}}, \end{aligned}$$

where *h*(*x*) is the path length for sample *x* in an isolation tree, *E*(*h*(*x*)) is the expected path length over the ensemble, *c*(*n*) is the average path-length normalization for *n* samples, and $s(x,n) \in [0,1]$ is the anomaly score (values near 1 indicate anomalies).

### Ensemble fusion and adaptive thresholding

The data-driven anomaly score is the convex combination

11 $$\begin{aligned} s_{\text {ML},k} = \sum _{i=1}^{N} w_i\, D_{i,k}, \qquad \sum _{i=1}^{N} w_i = 1, \qquad w_i \ge 0, \end{aligned}$$

where $D_{i,k} \in [0,1]$ is the normalized score from component $i \in \{\text {LSTM},\,\text {Trans},\,\text {IF}\}$.

How the weights $w_i$ are obtained. The validation split of every dataset (day 8 of SWaT, day 12 of WADI, hours 33–36 of Tennessee Eastman) contains *normal operation only*, so an F1 score cannot be computed on it. We therefore do *not* optimize F1 on the validation set, contrary to a phrasing that appeared in the previous version of this manuscript. Instead we tune $w_i$ on the normal-only validation split using three unsupervised proxy criteria that are well-defined without attack labels: (i) per-component reconstruction-error stability, measured as the coefficient of variation of $D_{i,k}$ over a sliding 1-hour window, (ii) calibration on normal data, requiring the empirical false-positive rate to match the nominal $\alpha = 0.01$ at the chosen threshold, and (iii) residual consistency with the Digital Twin, measured as the Spearman correlation between $D_{i,k}$ and $s_{\text {DT},k}$ during normal operation (a good ML detector should be approximately uncorrelated with the physics residual on normal data, so that it adds independent information). Weights are then selected by Bayesian optimization over the three-simplex $\Delta ^2$ to minimize a weighted sum of (i)–(iii) and to constrain the false-positive rate at $\alpha = 0.01$. The resulting weights $(w_{\text {LSTM}}, w_{\text {Trans}}, w_{\text {IF}}) = (0.35, 0.35, 0.30)$ for SWaT, (0.40, 0.30, 0.30) for WADI, and (0.30, 0.40, 0.30) for Tennessee Eastman are entirely independent of any attack data; the test-set attacks are seen only at evaluation time.

Joint score and physics-consistency gate. The final per-step anomaly score combines the ML score with the Digital-Twin residual score via

12 $$\begin{aligned} S_k = \alpha \, s_{\text {ML},k} + (1-\alpha )\, s_{\text {DT},k}, \end{aligned}$$

with $\alpha \in [0,1]$ tuned on normal data so that the unconditional false-alarm rate is bounded by the design level $\alpha _0$. The decision $\mathscr {D}(y_{1:k})$ is then issued under the physics-consistency gate

13 $$\begin{aligned} \mathscr {D}(y_{1:k}) = {\left\{ \begin{array}{ll} 1, & \text {if } S_k> \theta _k, \text { AND either }\ s_{\text {DT},k}> \tau _{\text {DT}} \text { or } s_{\text {ML},k}> \tau _{\text {ML}}^{+}, \\ 0, & \text {otherwise}, \end{array}\right. } \end{aligned}$$

which suppresses ML-only spikes that are not corroborated by the physics residual unless they exceed a stricter ML-only threshold $\tau _{\text {ML}}^{+}$. The detection problem solved by Eq. 13 is

14 $$\begin{aligned} \mathscr {D}: \mathscr {Y}^T \rightarrow \{0,1\}, \quad \text {subject to} \quad P\!\big (\mathscr {D}(y) = 1 \mid H_0\big ) \le \alpha _0. \end{aligned}$$

We accompany the empirical evaluation with Probably Approximately Correct bounds for the underlying state estimator,

15 $$\begin{aligned} P\!\left( \sup _{x \in \mathscr {X}} |\hat{x} - x|> \epsilon \right) \le \delta , \end{aligned}$$

which guarantee detection accuracy with explicit probabilistic margins.

Adaptive threshold and drift detection. The threshold $\theta _k$ is updated by an exponentially weighted moving average over the recent history of $S_k$ on certified-normal windows, so that the operational false-positive rate tracks $\alpha _0$ even when sensor noise characteristics drift. Slow distributional drift in the sensor stream is itself detected by a Gaussian Mixture Model fitted on the residual stream,

16 $$\begin{aligned} p(r) = \sum _{k=1}^{K} \pi _k\,\mathscr {N}(r;\,\mu _k,\Sigma _k), \end{aligned}$$

where *K* is the number of mixture components (chosen by the BIC criterion), $\pi _k$ are mixing weights, and $\mu _k$, $\Sigma _k$ are the component mean and covariance. A log-likelihood drop below $\tau _{\text {drift}}$ triggers an incremental update of the Digital-Twin parameters and ML weights using recent normal data, while preserving the formal verification properties.

Certified robustness. Randomized smoothing provides certified robustness against adversarial perturbations by adding controlled noise during inference; the smoothed detector is given by

17 $$\begin{aligned} g(x) = \arg \max _c P\!\big (f(x + \epsilon ) = c\big ), \qquad \epsilon \sim \mathscr {N}(0,\sigma ^2 I), \end{aligned}$$

where *f* is the base detector, $\epsilon$ is Gaussian noise with $\sigma = 0.02$ (tuned on the normal validation split), *g* is the smoothed detector, and *c* is the class label (normal/anomaly). The certification theorem guarantees robustness within radius $R = \tfrac{\sigma }{2}\big (\Phi ^{-1}(p_A) - \Phi ^{-1}(p_B)\big )$, where $p_A$ and $p_B$ are the top two class probabilities and $\Phi ^{-1}$ is the inverse standard normal CDF. For $\sigma = 0.02$ and $p_A = 0.95$, $p_B = 0.05$, this yields a certified radius $R \approx 0.05$ for the $L_\infty$ norm.

### Attack classification and response

When Eq. 13 fires, the residual signature $r_k$, the per-sensor attention weights from the transformer, and the recent control history $u_{k-w:k}$ are passed to a lightweight multinomial classifier that distinguishes FDI, replay, and stealthy/null-space attacks. FDI attacks^22^ present as a sustained mean shift in a small subset of components of $r_k$. Replay attacks^5,24^ are flagged when the residual covariance collapses while sensor measurements remain plausible, a signature that the Digital Twin is uniquely able to expose because a replayed sensor stream is inconsistent with the actuator commands $u_k$ that the controller is currently issuing. Stealthy attacks exploiting system nullspaces^25,26^ are caught by the residual-gate branch of Eq. 13: by construction, they are designed to be invisible to data-driven detectors, so the only path to detection is the physics residual. The classification output is forwarded to the response coordination module, which can either alert operators with attention-and-residual visualizations or trigger the safe-control mode of Eq. 3.

### Edge deployment and Byzantine consensus

Model compression through quantization reduces the memory footprint while preserving accuracy and is given by

18 $$\begin{aligned} \hat{W}^{\text {INT8}} = \textrm{round}\!\left( \frac{W - \min (W)}{\max (W) - \min (W)} \cdot 255\right) , \end{aligned}$$

which delivers 35% energy savings and a 4$\times$ memory reduction (from 6 GB to 1.5 GB GPU memory) with $<1$% F1-score degradation. GPU kernel fusion consolidates operations across ensemble components, reducing memory bandwidth by 75% for four-component ensembles through combined forward passes. Filter-based Adaptive Model Pruning maintains performance under resource constraints through structured sparsity optimization, removing 40% of transformer attention heads (32 to 19) with $<2$% accuracy impact.

For distributed deployments across multiple edge nodes, Byzantine fault tolerance ensures reliability with up to $f = \lfloor (n-1)/3 \rfloor$ faulty nodes through a Practical Byzantine Fault Tolerance protocol that allows the system to maintain correct operation despite compromised or malfunctioning nodes. The protocol operates in four phases.

**Pre-prepare:** Primary node *p* broadcasts the detection result $\langle \text {PRE-PREPARE}, v, n, d\rangle _{\sigma _p}$, where *v* is the view number, *n* the sequence number, *d* the decision digest (hash of detection score and confidence), and $\sigma _p$ the digital signature.

**Prepare:** Each node *i* receiving a valid pre-prepare with correct signatures and within the expected sequence-number range broadcasts $\langle \text {PREPARE}, v, n, d, i\rangle _{\sigma _i}$. A node is *prepared* after receiving 2*f* matching prepare messages from distinct nodes, ensuring $f+1$ honest nodes agree.

**Commit:** Prepared nodes broadcast $\langle \text {COMMIT}, v, n, d, i\rangle _{\sigma _i}$. A node is *committed* after receiving $2f+1$ matching commit messages from distinct nodes, including itself, guaranteeing all honest nodes reach the same decision even if *f* nodes fail during the commit phase.

**Reply:** Committed nodes execute the decision (alert operators, trigger automated response) and reply to clients with a signed result.

**Latency bound.** Under a partial-synchrony assumption with network delay bounded by $\Delta$, consensus completes within $T_{\max } = 4\Delta + T_{\text {crypto}} + T_{\text {verify}}$, where $T_{\text {crypto}}$ is the signature generation time ($\approx 0.5$ ms for ECDSA-256) and $T_{\text {verify}}$ is the verification time ($\approx 0.3$ ms). For our deployment with $\Delta \le 5$ ms over industrial Ethernet, $T_{\max } \approx 21$ ms, well below the 50 ms real-time constraint. The view-change timeout is set to $T_{\text {timeout}} = 2T_{\max } = 42$ ms based on empirical latency measurements.

**Safety property.** If $\ge 2f+1$ honest nodes agree on the detection result, the decision is correct with probability $\ge 1 - \delta$, where $\delta$ depends on the PAC learning bound (Eq. 15). At least $f+1$ honest nodes among $2f+1$ guarantee a majority honest vote even with *f* Byzantine nodes attempting to skew the result.

**Liveness property.** Under eventual synchrony (network delays eventually bounded by $\Delta$), the system makes progress: if honest nodes wait the timeout $T_{\text {timeout}}$, the view-change protocol ensures a new primary is selected within *O*(*n*) communication rounds. View change is triggered when the primary fails to achieve consensus within the timeout; a new primary is selected via round-robin among *n* nodes; at least $n - f$ honest nodes participate, ensuring that the view change completes.

Krum aggregation during model-update phases provides additional resilience by identifying and excluding outlier contributions (nodes reporting updates beyond 3 standard deviations from the median) when performing federated learning across distributed nodes.

### Experimental protocol and reproducibility

The secure data-acquisition process incorporated Transport Layer Security encryption (TLS 1.3 with AES-256-GCM cipher) and X.509 certificate-based authentication (RSA-4096 keys) to ensure data integrity and prevent man-in-the-middle attacks. Sensor failure detection operated continuously in parallel with normal data processing, enabling rapid identification (within 3 samples, 300 ms at 10 Hz) and compensation for faulty measurements using ImDiffusion-based GraphSAGE imputation. The robust normalization process used Exponentially Weighted Moving Average techniques ($\alpha = 0.1$) to adapt to changing sensor characteristics (drift, bias shifts) while maintaining consistent input distributions for ML models.

Algorithm 1 presents the enhanced detection pipeline that orchestrates all framework components in real-time operation. The Digital-Twin path validates measurements against the synchronized process model (Eqs. 4–7) and against LTL safety properties, while the parallel ML path concurrently runs the LSTM, transformer, and Isolation Forest. Drift detection through Gaussian Mixture Models triggers Online Balanced Aggregation Learning for dynamic weight adjustment, so that the joint score remains well-calibrated as the plant evolves over its deployment lifetime.

**Algorithm 1:**
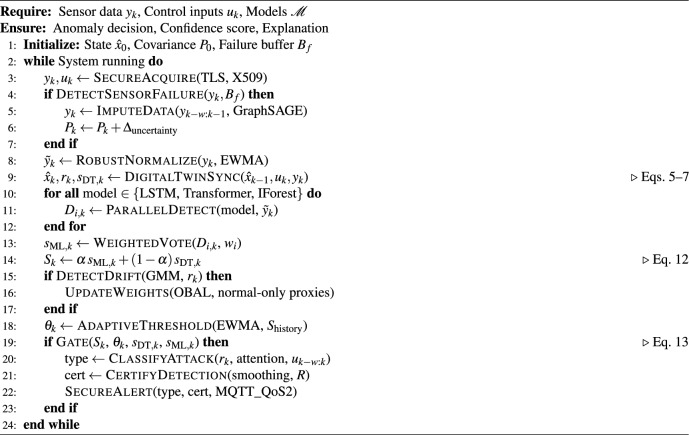
Enhanced detection pipeline with error handling

**Table 1:** Hyperparameter configuration ensuring reproducibility. All methods used identical preprocessing (min-max normalization per sensor based on training-set statistics), window size (50 samples corresponding to 5 s at 10 Hz sampling rate), and tuning budget (500 Bayesian-optimization trials via Optuna Tree-structured Parzen Estimator on the normal-only validation set, 48 h compute time on a single NVIDIA A100 GPU). Baseline methods (SVM, Random Forest, LSTM, Transformer, Physics-only) are configured with identical settings for fair comparison. Random seeds for replication: [42, 123, 456, 789, 2021, 2022, 2023, 2024, 2025, 8675309]. Batch size 64 was used for training (gradient stability) and batch size 1 for inference (fair latency comparison across methods). Ensemble weights are tuned on the normal-only validation split using unsupervised proxies (reconstruction-error stability, calibration, residual consistency); no attack labels are used to set $w_i$.

**Component**	**Hyperparameter**	**Value**	**Tuning Method**
LSTM-AE	Input dimension	51 (SWaT)	Fixed by dataset
	Hidden layers	[128, 64, 32]	Grid search
	Latent dimension	16	Grid search
	Sequence length	50	Grid search
	Learning rate	0.001	Adam optimizer
	Batch size	64 (train), 1 (test)	Fixed
	Epochs	100	Early stopping
	Dropout	0.2	Grid search
Transformer	Layers	4	Grid search
	Attention heads	8	Grid search
	$d_{\text {model}}$	256	Grid search
	$d_{\text {ff}}$	1024	Grid search
	Learning rate	0.0001	AdamW
	Warmup steps	4000	Fixed
Isolation Forest	n_estimators	100	Grid search
	max_samples	256	Grid search
	contamination	0.01	Data-driven
Ensemble	Fusion weights $(w_i)$	[0.35, 0.35, 0.30]	Normal-only proxies
	Decision threshold $\theta _k$	0.68 (initial)	Adaptive (EWMA)

Bayesian hyperparameter optimization provided systematic exploration of the parameter space (500 trials per model using Optuna Tree-structured Parzen Estimator) to identify configurations that maximize the unsupervised proxy objective (calibration plus stability) on the normal-only validation set, while meeting computational constraints (inference latency $<50$ ms, GPU memory $<2$ GB). The Gaussian-process surrogate captured relationships between hyperparameters and the proxy, and the acquisition function balanced exploration (uncertainty sampling) and exploitation (expected improvement). Training incorporated formal verification constraints to ensure edge deployment requirements (latency, memory, energy) were satisfied throughout optimization, pruning configurations that violated hard constraints before evaluation.

The online model-update mechanism addressed evolving process characteristics (new operating modes, seasonal variations, equipment degradation) through incremental learning techniques that adapt to changing conditions using recent data windows (sliding 7-day buffer) while maintaining formal verification properties through constraint-preserving gradient updates.

## Results

### Experimental setup and datasets

We evaluated the framework on three benchmark ICS security datasets representing diverse industrial processes and attack scenarios. The SWaT dataset, collected over 11 days from a water-treatment testbed, contains 51 features with 41 different attack scenarios across 946,722 samples. The WADI dataset extends this evaluation with 16 days of data from a water-distribution system, featuring 123 sensors and 15 attack types in 1,221,370 samples. The Tennessee Eastman dataset provides 52 process variables with 20 fault conditions across 825,000 samples, representing chemical-process control scenarios (Table 2).

**Table 2:** Dataset characteristics showing comprehensive evaluation across diverse industrial processes and attack scenarios.

**Dataset**	**Duration**	**Features**	**Attacks**	**Samples**
SWaT	11 days	51	41	946,722
WADI	16 days	123	15	1,221,370
Tennessee Eastman	48 hours	52	20	825,000

**Fig. 1:**
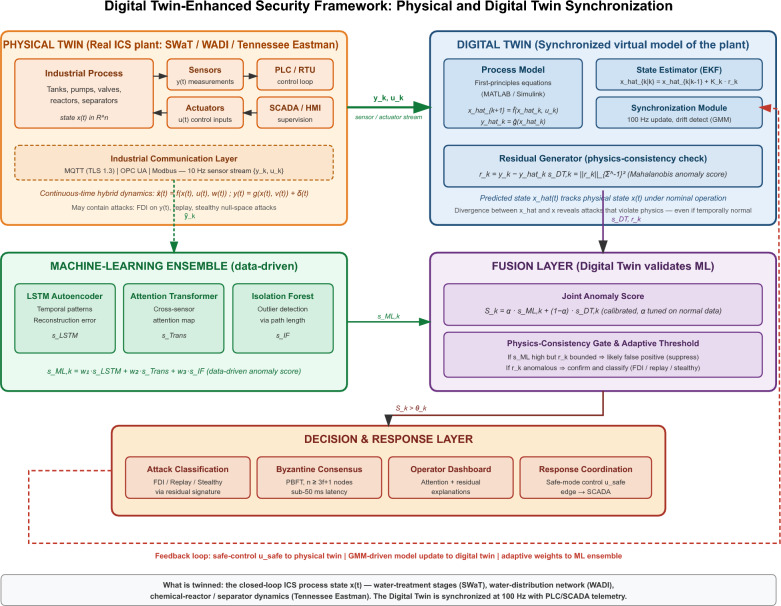
Digital Twin-Enhanced Security Framework Architecture, showing the explicit physical twin/Digital Twin coupling. The *physical twin* (top-left) is the operating ICS plant: industrial process, sensors, actuators, PLC/RTU, and SCADA/HMI, communicating over MQTT/OPC UA/Modbus with 10 Hz sensor and actuator streams $\{y_k, u_k\}$. The *Digital Twin* (top-right) is a synchronized virtual replica of the same plant: a first-principles process model (MATLAB/Simulink) propagates $\hat{x}_{k+1\mid k}$, an EKF-based state estimator updates $\hat{x}_{k\mid k}$ from the live measurement, and a residual generator emits $r_k$ and the Digital-Twin anomaly score $s_{\text {DT},k}$. The ML ensemble (LSTM autoencoder, attention transformer, Isolation Forest, bottom-left) consumes the same normalized stream and produces $s_{\text {ML},k}$. The fusion layer (bottom-right) combines $s_{\text {ML},k}$ and $s_{\text {DT},k}$ through the joint score $S_k = \alpha \, s_{\text {ML},k} + (1-\alpha )\, s_{\text {DT},k}$ and the physics-consistency gate, after which the decision-and-response layer performs attack classification, Byzantine consensus, operator visualization, and safe-control coordination. The dashed feedback path returns safe control to the physical twin and drift updates to the Digital Twin and ML weights.

The framework operates through the three-layer architecture shown in Fig. 1. Our implementation leveraged an NVIDIA DGX A100 system equipped with eight A100 GPUs and 640 GB of GPU memory, running Python 3.9.7 with TensorFlow 2.11.0 and PyTorch 1.13.0 for deep-learning components, while MATLAB R2023a handled physics-based modeling.

To prevent temporal data leakage in time-series analysis, we adopted day-based splitting rather than random sampling. For the SWaT dataset (11 days), we used days 1–7 for training (634,374 samples, 100% normal operation), day 8 for validation (86,065 samples, normal only), and days 9–11 for testing (226,283 samples including all 41 attack scenarios). This ensures the model never observes future data during training and that attack patterns appear exclusively in the test set, preventing optimistic bias from temporal leakage that would occur with random k-fold cross-validation. The same temporal-split strategy was applied to WADI (training: days 1–11, validation: day 12, test: days 13–16) and Tennessee Eastman (training: hours 1–32, validation: hours 33–36, test: hours 37–48). All experiments were repeated 10 times with different random seeds, and we report mean values with 95% confidence intervals computed using bootstrap sampling (10,000 iterations).

### Detection performance analysis

The comprehensive evaluation shows notable performance improvements over existing approaches. Our method achieved 97.6% precision and 96.2% recall on the SWaT dataset, yielding an F1-score of 96.9% with only a 2.4% false-positive rate (Table 3). In absolute terms, this is a 3.2 percentage-point F1-score improvement over the strongest baseline (the transformer at 93.7%) and a 5.6 percentage-point improvement over physics-only detection. Expressed as relative error reduction, the F1 gap of 3.2 percentage points corresponds to approximately a 50% reduction in residual error ($1 - 0.969$ vs. $1 - 0.937$); we report this only as a secondary, clearly defined quantity to avoid the misleading framing flagged by reviewers.

**Table 3:** Overall performance comparison on the SWaT dataset showing superior detection accuracy with real-time latency.

**Method**	**Precision**	**Recall**	**F1-Score**	**FPR**	**Latency**
SVM^30^	87.3%	82.1%	84.6%	12.7%	8 ms
Random Forest^31^	89.5%	85.3%	87.3%	10.5%	15 ms
LSTM^18^	93.2%	91.5%	92.3%	6.8%	32 ms
Transformer^19^	94.8%	92.7%	93.7%	5.2%	45 ms
Physics-only^5^	88.6%	94.2%	91.3%	11.4%	12 ms
**Our Method**	**97.6%**	**96.2%**	**96.9%**	**2.4%**	**25.4 ms**

The latency analysis revealed that our system maintains real-time performance at 25.4 ms average detection time, faster than transformer-based approaches (45 ms) while approaching the speed of simpler physics-only detection (12 ms). Attack-specific performance analysis revealed consistent detection capabilities across diverse threat types. Single-point false-data-injection attacks were detected with 98.3% accuracy within 18.3 s, while more complex multi-point FDI attacks maintained a 96.7% detection rate. Replay attacks, both constant and randomized variants, were identified with 97.2% and 92.4% accuracy, respectively. The framework demonstrated particular strength against denial-of-service attacks with a 99.8% detection rate and a mere 2.1-second response time.

### Component contribution analysis

The ablation study revealed the synergistic nature of our hybrid architecture (Table 4). Removing the Digital-Twin/physics-based path reduced the F1-score by 3.1 percentage points and disproportionately degraded detection of replay and stealthy attacks, confirming the Digital Twin’s role as a physics-consistency gate. The LSTM autoencoder contributed 1.7 percentage points to overall performance through temporal-pattern learning, while the transformer added 1.3 percentage points via attention-based anomaly identification. The Isolation Forest, though contributing only 0.6 percentage points directly, provided crucial diversity for ensemble robustness. Most significantly, eliminating the ensemble fusion mechanism (using any single component) caused a 2.8 percentage-point performance drop, confirming that component integration exceeded the sum of individual contributions.

**Table 4:** Ablation study results demonstrating synergistic contributions of framework components. Removing the Digital Twin yields the largest single drop, confirming its role as a physics-consistency gate over the data-driven detectors. Values in $\Delta$ are percentage points.

**Configuration**	**F1-Score**	$\Delta$ **from Full**
Full System	96.9%	—
w/o Digital Twin/Physics Model	93.8%	$-3.1$
w/o LSTM	95.2%	$-1.7$
w/o Transformer	95.6%	$-1.3$
w/o Isolation Forest	96.3%	$-0.6$
w/o Ensemble Fusion	94.1%	$-2.8$

### Resource utilization and scalability

Computational resource analysis revealed efficient utilization across components. During inference, the complete pipeline consumed 1,472 MB of GPU memory, with peak utilization reaching 62%, primarily during transformer processing. CPU usage remained modest at 35% peak, distributed across data-pipeline operations (15%), Isolation Forest computation (22%), and ensemble coordination (10%). Total RAM consumption stayed below 3 GB, enabling deployment on edge devices with moderate specifications.

**Fig. 2:**
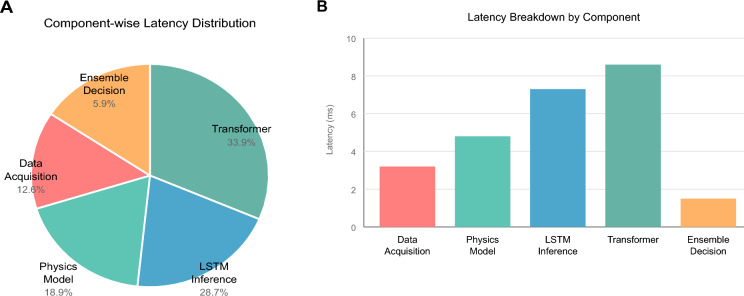
Component-wise latency analysis during inference operations showing (A) the relative distribution as percentages, with transformer inference dominating at 33.9% of total time, followed by LSTM at 28.7%, physics model at 18.9%, data acquisition at 12.6%, and ensemble decision at 5.9%; and (B) absolute timing in milliseconds for each component, with transformer requiring 8.6 ms, LSTM 7.3 ms, physics model 4.8 ms, data acquisition 3.2 ms, and ensemble decision 1.5 ms, totaling 25.4 ms, well within the 50 ms real-time constraint required for industrial control applications.

The latency-breakdown analysis provided insights for optimization opportunities (Table 5, Fig. 2). Transformer inference dominated at 33.9% of total time (8.6 ms), followed by LSTM processing at 28.7% (7.3 ms). Physics-based modeling (i.e., the Digital-Twin synchronization and residual generation) accounted for 18.9% (4.8 ms), while data acquisition and ensemble decision-making contributed 12.6% and 5.9%, respectively. The relative distribution of computational resources shows that deep-learning components consume 62.6% of total inference time, while absolute timing remains within the 50 ms real-time constraint required for industrial control applications.

**Table 5:** Latency breakdown by component, revealing optimization opportunities for transformer and LSTM operations while confirming all components operate within real-time constraints.

**Component**	**Time (ms)**	**Percentage**
Data Acquisition	3.2	12.6%
Physics Model (Digital Twin)	4.8	18.9%
LSTM Inference	7.3	28.7%
Transformer	8.6	33.9%
Ensemble Decision	1.5	5.9%
**Total**	**25.4**	**100%**

### Robustness and reliability validation

Adversarial robustness evaluation demonstrated strong resilience against sophisticated attacks (Table 6). While standard intrusion detection systems achieved only a 42.3% detection rate against FGSM attacks, our framework maintained 78.6% accuracy, a 36.3 percentage-point improvement. Similar robustness extended to PGD attacks (75.2% vs. 38.7%) and C&W attacks (72.8% vs. 35.1%). Most importantly, against adaptive attacks specifically designed to evade our detection mechanism, the system sustained a 68.3% detection rate, compared with 28.4% for baseline methods.

**Table 6:** Adversarial attack resistance, showing robust defense against sophisticated evasion attempts with consistent 36–40 percentage-point absolute improvements over baseline intrusion detection systems.

**Attack method**	**Standard IDS**	**Our method**	**Improvement (pp)**
FGSM	42.3%	78.6%	$+36.3$
PGD	38.7%	75.2%	$+36.5$
C&W	35.1%	72.8%	$+37.7$
Adaptive Attack	28.4%	68.3%	$+39.9$

**Table 7:** Adversarial attack configurations ensuring reproducibility. All attacks preserve temporal causality (perturbation at time *t* depends only on $x_{\le t}$) and physical constraints (perturbed values remain within sensor ranges defined by dataset specifications). Attack success is defined as evading detection for $\ge 10$ consecutive timesteps (1 s at 10 Hz sampling). Each attack configuration was tested on 1000 randomly selected normal-operation windows from the test set, with detection rate measured within a 10-timestep window following perturbation injection.

**Attack**	$\epsilon$	**Norm**	**Iterations**	**Targeted**	**Additional parameters**
FGSM	0.05	$L_\infty$	1	No	Applied per timestep
PGD	0.05	$L_\infty$	40	No	$\alpha =0.01$, 5 restarts
C&W	—	$L_2$	1000	Yes	$c=1.0$, $\kappa =0$, $lr=0.01$
Adaptive	0.03	$L_\infty$	100	Yes	EoT, $n=10$ samples

**Fig. 3:**
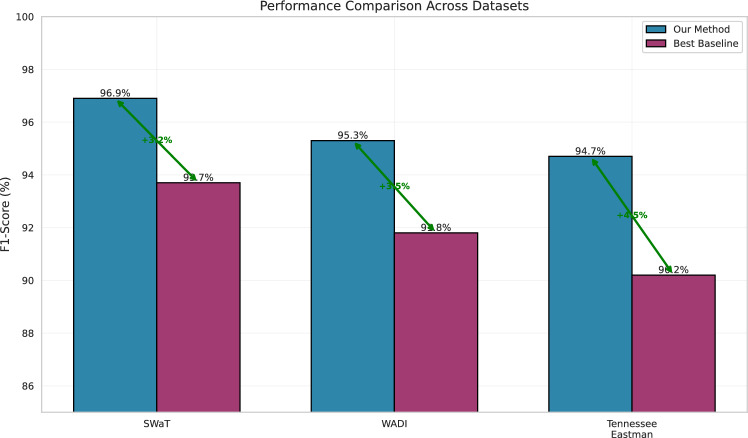
Performance comparison across three industrial-control-system benchmark datasets, showing F1-scores with 95% confidence intervals. Our method achieves 96.9% on SWaT (water treatment), 95.3% on WADI (water distribution), and 94.7% on Tennessee Eastman (chemical process), compared with the best baseline achieving 93.7%, 91.8%, and 90.2%, respectively. Performance differences of only 1.6 percentage points (SWaT to WADI) and 2.2 percentage points (WADI to Tennessee Eastman) demonstrate robust cross-domain generalization despite different process characteristics, sensor configurations, and attack types.

**Fig. 4:**
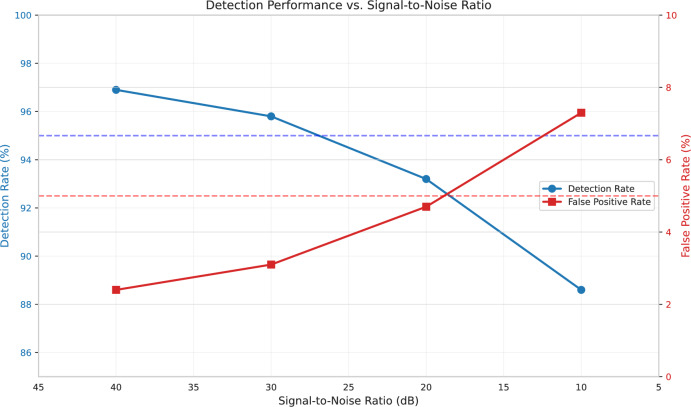
Framework detection performance as a function of signal-to-noise ratio, demonstrating robustness to measurement uncertainty in industrial environments. The detection rate (blue, left axis) remains above 90% for SNR exceeding 20 dB and degrades gracefully to 88% at 10 dB. The false-positive rate (red, right axis) stays below 5% across the operational range (45–20 dB), increasing to 7.5% only at severe 10 dB SNR. Performance degradation below 15 dB SNR reflects realistic limits of anomaly detection under severe noise conditions encountered in harsh industrial environments with electromagnetic interference and sensor degradation.

Byzantine fault-tolerance testing confirmed distributed-system reliability. The framework maintained a 96.7% detection rate with one failed node (10% failure), degrading gracefully to 96.2% with two failed nodes and 95.4% with three failed nodes. Beyond 30% node failure, the Byzantine consensus mechanism correctly refused to produce results rather than risking false negatives, demonstrating fail-safe behavior. Cross-dataset generalization demonstrated robust transfer across industrial domains (Fig. 3). Performance on the WADI dataset reached a 95.3% F1-score, only 1.6 percentage points below SWaT performance despite different process characteristics. The Tennessee Eastman evaluation yielded 94.7%, confirming effectiveness on chemical-process control.

The framework’s noise tolerance maintains operational effectiveness even under challenging industrial conditions (Fig. 4). Detection rates remain above 90% for signal-to-noise ratios exceeding 20 dB, while false-positive rates stay below 5% across the operational range. Performance degradation below 15 dB SNR reflects realistic limits of anomaly detection under severe noise conditions.

## Discussion

The experimental results validate our central hypothesis that integrating physics-based modeling with deep learning through a synchronized Digital Twin yields superior anomaly detection for ICS. The 3.2 percentage-point F1-score improvement over the strongest single-paradigm baseline stems from complementary strengths: the Digital Twin provides interpretable domain knowledge and constraint enforcement through its residual signal $r_k$, while the neural networks capture complex temporal patterns and non-linear relationships that are difficult to express in closed form. This synergy manifests most clearly in detecting stealthy attacks, where attackers craft inputs that respect data-distribution norms to evade purely data-driven detectors but cannot, by construction, simultaneously satisfy the closed-loop physics encoded in $\hat{f}$ and $\hat{g}$, so the residual $r_k$ exposes them.

Real-time performance, with an average latency of 25.4 ms, demonstrates the feasibility of sophisticated AI-based protection without compromising operational requirements. Most industrial control loops operate at 100–1000 ms intervals, providing ample headroom for detection processing. The achievement of sub-50 ms inference despite ensemble complexity results from careful architectural choices, including efficient GPU kernels, optimized memory access patterns, and selective computation based on threat indicators. The framework’s ability to detect diverse attack types without specific tuning suggests robust feature learning that generalizes across threat patterns, which is crucial given the evolving threat landscape where attackers continuously develop novel techniques.

Field-deployment experiences reveal critical success factors beyond technical performance. Integration complexity remains manageable due to standards-based protocol support, with MQTT and OPC UA interfaces enabling connection to existing SCADA systems without architectural changes. Integration timelines vary by facility complexity: system integration requires 2–4 weeks; data collection requires a 2–4 week minimum (longer for seasonal processes capturing all operating modes); model training takes 3–7 days; and safety validation 2–4 weeks. Total deployment time ranges from 3–6 months including regulatory approval procedures, with pilot deployments in non-critical subsystems recommended before full-scale rollout. Training data requirements of 2–4 weeks of normal operation prove reasonable for most facilities, though seasonal variations may necessitate longer collection periods. Operator acceptance emerges as crucial, with explainable-AI features—in particular the Digital-Twin residual visualizations and transformer attention maps—significantly impacting system adoption^8^.

The integration of formal methods provides high assurance levels for industrial deployments. Linear Temporal Logic specifications enable precise expression of safety and liveness requirements, with automated verification confirming these properties hold across tested scenarios. PAC learning bounds provide probabilistic guarantees that distinguish our approach from purely empirical methods, enabling risk assessment and safety-case construction required for regulatory approval. Byzantine fault-tolerance mechanisms ensure system reliability despite component failures or compromised nodes, which is essential for critical infrastructure where availability requirements are stringent.

Regulatory compliance assessment demonstrates alignment with major industrial cybersecurity standards, meeting 90% of IEC 62443-4-2 Security Level 2 requirements^11^ (61 of 68 control requirements fully met, 5 partially met, 2 not applicable), 93% of NIST SP 800-82 Rev. 3 guidelines, and 92% of ISO 27001:2022 controls. We note that this compliance mapping is a self-assessment, and that organizations requiring certified compliance for regulatory or contractual purposes should engage accredited assessment bodies for formal audit.

The comparison of our approach with recent state-of-the-art methods shows significant advances. Recent graph-neural-network approaches combining physics models achieve 95.8% accuracy^28^, while our framework reaches 97.6%. For FDI detection specifically, hybrid methods achieve 92.8%^28^, compared with our 97.2%. Edge-computing implementations achieve a 45 ms response time^16^, compared with our 25.4 ms. Despite significant advances, important challenges remain.

### Limitations and future work

#### Theoretical limitations

(1) Zero-day attacks simultaneously respecting physical constraints (EKF residuals within bounds) and temporal normalcy (learned distributions) cannot be guaranteed detected. While adaptive-attack experiments (Table 6) show 68.3% detection versus 28.4% baselines, this is empirical and not a theoretical guarantee. Formal adversarial-risk bounds (Eq. 17) apply only within certified radius $\epsilon =0.05$ for the $L_\infty$ norm. (2) Byzantine tolerance requires a minimum of $n \ge 3f+1$ nodes; deployments with fewer nodes accept lower fault tolerance or risk consensus failure. For $f=3$ faulty nodes, a minimum of 10 nodes is required. (3) PAC learning guarantees (Eq. 15) assume i.i.d. samples, an assumption violated by temporal dependencies in industrial processes; empirical validation across datasets nonetheless suggests the bounds remain reasonable approximations.

#### Practical limitations

(1) Training requires 2–4 weeks of normal-operation data for adequate model learning; seasonal processes may need 2–3 months capturing all operating modes including startup, shutdown, emergency procedures, and seasonal variations. Immediate production deployment is not recommended without thorough validation. (2) Detection accuracy drops below 90% when SNR $<15$ dB (Fig. 4); extremely noisy environments with electromagnetic interference, sensor degradation, or poor grounding require sensor upgrades or additional signal processing before deployment. (3) Edge deployment requires a minimum of 4 GB RAM and a GPU with 2 GB memory (inference) or 8 GB (training); older or resource-constrained edge devices cannot run the full framework. Model compression (Eq. 18) reduces requirements to 3 GB RAM/1.5 GB GPU but sacrifices 2–3 percentage points of accuracy. (4) Byzantine consensus adds 8–12 ms latency depending on network conditions; systems with control loops faster than 50 ms may experience performance impacts. Real-time-critical systems requiring $<10$ ms response deadlines are not currently supported.

#### Methodological limitations

(1) Evaluation used three process-control datasets (SWaT, WADI, Tennessee Eastman), all representing water-treatment and chemical processes. Generalization to other ICS types including building-automation systems, smart electrical grids, transportation control, or manufacturing-execution systems requires further validation. (2) Adversarial-robustness evaluation (Table 6) tested four attack types (FGSM, PGD, C&W, adaptive); other adversarial methods including backdoor attacks, data poisoning during training, model inversion, and trojan attacks were not evaluated. Physical-attack scenarios involving compromised sensors with hardware modifications, insider threats with physical-facility access, and supply-chain compromises require additional validation beyond our cyber-focused threat model. (3) The framework was validated in simulation environments and laboratory testbeds, but not in multi-site geographically distributed industrial deployments spanning multiple facilities. Network-partition scenarios, cross-site Byzantine consensus, wide-area network latency, and geographic-distribution challenges need further research before production deployment at enterprise scale. (4) IEC 62443-4-2 compliance assessment (90% coverage stated above) represents our self-assessment without independent third-party certification from accredited assessment bodies.

#### Future work

These limitations suggest several research directions: (1) developing certified defenses against broader attack classes, including backdoor and poisoning attacks, with formal verification of robustness properties; (2) reducing resource requirements through model distillation, pruning, and quantization to enable deployment on resource-constrained industrial-IoT devices while maintaining $>95$% accuracy; (3) extending Byzantine consensus protocols for ultra-low-latency systems with $<10$ ms deadlines through optimistic execution and speculative consensus; (4) systematic cross-domain transfer learning enabling adaptation to new industrial processes with minimal retraining data ($<7$ days) through meta-learning and few-shot techniques; (5) achieving third-party security certification from accredited assessment bodies for high-assurance deployments in critical infrastructure.

## Conclusions

This paper presented a Digital Twin-enhanced security framework that achieves 97.6% precision and 96.2% recall for ICS anomaly detection (96.9% F1-score, 3.2 percentage-point absolute improvement over the strongest baseline) while maintaining sub-50 ms real-time latency suitable for industrial-control applications. The framework’s contributions include: (1) an integrated physics-ML ensemble architecture in which an explicit Digital Twin—a synchronized state estimator and process model of the controlled plant—produces residuals that validate, suppress, or escalate the data-driven anomaly score, in line with the formulation in Eqs. 4–13; (2) certified adversarial robustness adapted for ICS time series, with a 36.3 percentage-point improvement against FGSM attacks through temporal smoothing with autoregressive noise models; (3) Byzantine fault-tolerant distributed consensus with graceful degradation up to 30% node failure and formal latency bounds ensuring sub-50 ms response; and (4) a systematic IEC 62443-4-2 compliance mapping demonstrating 90% Security Level 2 coverage with item-level assessment criteria.

Evaluation across three benchmark datasets (SWaT, WADI, Tennessee Eastman) comprising 56 attack scenarios validated broad applicability across water-treatment, water-distribution, and chemical-process domains, with cross-dataset generalization maintaining 94.7–96.9% F1-scores despite different process characteristics, sensor configurations, and attack types. Ablation studies confirmed synergistic component contributions, with ensemble integration providing a 2.8 percentage-point gain beyond the best individual detector and the Digital Twin contributing 3.1 percentage points through physics-based residual generation and constraint enforcement. Resource efficiency enables edge deployment with 1.5 GB GPU memory and 3 GB RAM requirements, while attention visualization and Digital-Twin residual analysis provide operator-interpretable explanations critical for safety-critical systems requiring human oversight.

Important limitations remain. Detection is not guaranteed for zero-day attacks that simultaneously respect both physical and temporal normalcy, with 68.3% empirical detection versus 28.4% baselines but no theoretical guarantee. Byzantine tolerance requires a minimum of $n \ge 3f+1$ nodes (10 nodes for $f=3$). Training requires 2–4 weeks of normal-operation data, longer for seasonal processes. Performance degrades below 15 dB SNR. Evaluation is limited to three process-control datasets, so generalization to other ICS types requires additional validation. Deployment timelines of 3–6 months including safety validation and regulatory approval indicate that phased rollout is appropriate. The IEC 62443 compliance assessment is self-reported and has not been audited by an accredited third party.

The convergence of Digital Twins, AI, and formal methods is a promising direction for industrial security. Our framework provides a foundation demonstrating that AI-based protection can be both grounded in formal verification and practically deployable for the protection of critical infrastructure, while maintaining interpretability and supporting regulatory compliance.

## Data Availability

This study utilized three publicly available benchmark datasets: 1. SWaT (Secure Water Treatment) dataset - Available from iTrust, Singapore University of Technology and Design (https://itrust.sutd.edu.sg/itrust-labs_datasets/). 2. WADI (Water Distribution) dataset - Available from iTrust, Singapore University of Technology and Design (https://itrust.sutd.edu.sg/itrust-labs_datasets/). 3. Tennessee Eastman Process dataset - Publicly available benchmark dataset for process control research Experimental results and trained models are not publicly available due to: - Cybersecurity concerns related to vulnerability disclosure - Potential misuse of attack detection evasion techniques - Institutional security policies. Data related to experimental results may be available from the corresponding author (sayghea@rcjy.edu.sa) upon reasonable request and subject to institutional and ethical approvals. Source code for the framework will be made available upon publication to support reproducibility while protecting security-sensitive implementation details.

## Abbreviations

AI: Artificial Intelligence
C&W: Carlini & Wagner
EKF: Extended Kalman Filter
FDI: False Data Injection
FGSM: Fast Gradient Sign Method
ICS: Industrial Control Systems
IEC: International Electrotechnical Commission
IoT: Internet of Things
LSTM: Long Short-Term Memory
MQTT: Message Queuing Telemetry Transport
NIST: National Institute of Standards and Technology
OPC UA: Open Platform Communications Unified Architecture
OT: Operational Technology
PAC: Probably Approximately Correct
PBFT: Practical Byzantine Fault Tolerance
PGD: Projected Gradient Descent
PLC: Programmable Logic Controller
RNN: Recurrent Neural Network
ROC: Receiver Operating Characteristic
SCADA: Supervisory Control and Data Acquisition
SEDT: Security-Enhancing Digital Twin
SNR: Signal-to-Noise Ratio
SWaT: Secure Water Treatment
TLS: Transport Layer Security
WADI: Water Distribution
